# Longevity and Developmental Stability in the Dung Fly *Sepsis cynipsea,* as Affected by the Ectoparasitic Mite, *Pediculoides mesembrinae*

**DOI:** 10.1673/031.009.6601

**Published:** 2009-12-03

**Authors:** Oliver Y. Martin, David J. Hosken

**Affiliations:** ^1^ Zoology Museum, University of Zurich, Winterthurerstrasse 190, CH-8057 Zurich, Switzerland; ^2^ Current address: ETH Zurich, Institute of Integrative Biology (IBZ), Universitätsstrasse 16, CH-8092 Zürich, Switzerland; ^3^ Current address: Centre for Ecology and Conservation, School of Biosciences, University of Exeter, Cornwall Campus, Penryn, Cornwall TR10 9EZ, United Kingdom

**Keywords:** fluctuating asymmetry, parasite, Diptera, Sepsidae, Phthiraptera, Menoponidae

## Abstract

Fluctuating asymmetry (FA) is a widely employed measure of developmental stability. It has been found to increase with many stressors including parasite infection. Associations between parasites and FA may exist for several reasons in addition to parasites being the direct cause of increased FA. Developmentally stable individuals may have superior immune systems, and be less susceptible to parasite infection, and/or may be less exposed to parasites than developmentally unstable ones. Mites negatively impact host fitness in a number of insects, and if FA is a reflection of general genetic quality, as has been proposed, associations between mite number and FA are predicted. Potential relationships were investigated between an ectoparasitic mite, *Pediculoides mesembrinae* (Canestrini) (Phthiraptera: Menoponidae) and FA in the common dung fly *Sepsis cynipsea* (L.) (Diptera: Sepsidae). While it was found that mite infested flies died much faster than flies without mites, indicating that mites indeed stress their hosts, counter to expectations, no associations between mites and FA were found in any analyses. Additionally, FA in mite-infected flies generally did not differ from previously published FA data from uninfected *S. cynipsea.* Nevertheless, parasitized males tended to be somewhat less asymmetrical than non-parasitized males, but based on our data, it does not appear that mite infestation is generally associated with developmental stability in *S. cynipsea.*

## Introduction

Bilaterally symmetrical traits should in principle be identical on either side of the body since they are under control of the same genes. However, deviations from perfect symmetry are common and these deviations are thought to convey information about developmental stability. Developmental stability, is the ability of a genotype to resist developmental perturbations, and is most frequently measured by fluctuating asymmetry (FA: small random deviations from perfect symmetry) (reviewed in Palmer 1994; Markow 1995; Møller and Swaddle 1997), although other types of symmetry may also convey information about developmental stability (Graham et al. 1998; Kark 2001).

Because of its presumed association with developmental stability, FA has been investigated in many contexts, including sexual selection (Møller 1992a; Eggert and Sakaluk 1994; Hunt and Simmons 1997; Tomkins and Simmons 1999; Gage 1998; Hosken 2001; Hosken et al. 2003; Tomkins and Simmons 2003), and associations between it and all manner of fitness measures have been reported (Stockley et al. 1996; Møller and Swaddle 1997; Palmer 2000). For example, Møller (1999) reported small but significant effects in meta-analyses of associations between FA and fecundity, survival and growth across studies. However, two more recent experimental studies found no associations between FA and these fitness measures (Martin and Hosken 2002; Woods et al. 2002). More generally, it was argued that FA reveals information on general genetic quality (reviewed in Møller and Swaddle 1997), although this idea remains contentious (Bjorksten et al. 2000). Nevertheless, what seems clear is that stress during ontogeny often increases FA (Waddington 1957; Parsons 1990; Hosken et al. 2000; reviewed in Hoffmann and Parsons 1991).

One stressor that frequently affects fitness and FA is parasitic infection (reviewed in Polak 1997b). This may be because parasites compete with their hosts for resources, and therefore impinge upon host metabolism, growth and development (Schall et al. 1982; Goater et al. 1993). Supporting this idea, some experimental manipulations of parasite loads provide evidence for a direct causal link between FA and parasites (Parsons 1990; Møller 1992b; Folstad et al. 1996). However, FA and parasites may be associated for several other reasons (Polak 1997b; Thomas et al. 1998). Firstly, heavily parasitized mothers may produce developmentally compromised offspring (Polak 1997a). Secondly, developmentally stable individuals may have superior immune systems, and hence be less susceptible to parasite infection (Polak 1993). For example, asymmetrical house flies, *Musca domestica,* were more likely to die from fungal infection, and experimental manipulations indicated this was because flies with high wing FA were more susceptible to infection (Møller 1996a). Thirdly, developmentally stable individuals may be less exposed to parasites than developmentally unstable ones (Hunt and Allen 1998). However, as with many FA associations, significant relationships between FA and parasites are not invariably found. For example, recent work by Ward et al. (1998) found no associations between various parasites and FA in six insect species.

The dung fly *Sepsis cynipsea* (Diptera: Sepsidae) is one of the most common dung pat inhabitants during northern summers (Parker 1972; Ward 1983). It has been the subject of two FA studies that provide different degrees of support for FA/fitness associations. Allen and Simmons (1996) found that fore-tibia FA was negatively associated with mating success, and Blanckenhorn et al. (1998) tested potential associations between FA and good genes. Among other things, they found no association between FA and growth rate or female fecundity, but a weak negative association with survival. Here associations were investigated between FA and the ectoparasitic mite, *Pediculoides mesembrinae* (Canestrini) (Phthiraptera: Menoponidae), in wild and laboratory reared flies. There is considerable evidence that mites are costly to their insect hosts. For example, Polak (1996) reported reduced fecundity in mite-infested flies, and in damselflies, mites negatively impact many fitness related traits and condition measures (reviewed in Yourth et al., 2002). *Pediculoides mesembrinae* is no exception, and is a true parasite that feeds on adult flies (H. Ochs personal communication). These mites are described as mainly parasitising Muscidae and Borboridae, frequently attacking larvae, pupae and adult flies, and all mite life stages are found on cow dung (H. Ochs personal communication). If FA generally reflects host quality, then associations with mite infection are predicted. To assess this supposition, we first tested if mites were costly to the flies, because a cost to parasitism would imply that they are stressful for their host, and FA and stress are thought to be intimately related. Potential associations between mite number and host FA were then tested to see if asymmetrical individuals are more prone to infection. Finally FA of mite-infected flies was compared with previously published FA measures from laboratory-reared, mite-free flies (Blanckenhorn et al. 1998) to see if mites *per se* increased host FA.

## Materials and Methods

### Longevity costs of mite infestation

To investigate the costs of mite infestation on the longevity of experimentally infected flies were compared with uninfected flies. Large (200ml) portions of dung, the egg laying and larval development substrate, were placed in *S. cynipsea* population cages and left overnight for females to lay eggs. These were then divided equally into mite-free and infected treatments. For the infected treatment, dung portions were infected with nymphs of the mite *P. mesembrinae* by placing large numbers of mites (ca. 100 per portion) on the dung surface. Mites hence had the opportunity to parasitize all fly life stages (egg, larvae, pupae, emerging imagines) until the eclosion of experimental adult flies. Dung from each treatment was then placed in a separate population cage (with sugar and water) under the same controlled climatic conditions until flies emerged. Emerging flies were sexed and checked for mites (mites swarm over pupae attaching themselves to freshly emerged flies). Ten randomly picked individuals of each sex infected with mites and the same number of uninfected flies control were retained for the experiment. Because this procedure was synchronized (i.e. flies from both treatments were collected on the same day) confounding effects of development time (which can affect the development of the immune system) can be ruled out, and no mites were found in the control cages. The experimental flies were placed singly in vials with ample sugar and water and checked daily for death. Longevity was recorded in days and once the flies died an estimate of body size was measured using length of the hind tibia length. Additionally, the number of mites attached to infected individuals was counted and used as a covariate in the analysis of longevity.

### Fluctuating asymmetry and mites

Adult flies collected from the field during summer 2000 to start a laboratory population were screened for the presence of mites. The initial collection was small and contained few infected flies (n = 11), and a larger collection was undertaken some weeks later (n = 76 flies with mites). These were pooled in subsequent analyses. Flies with mites were frozen at -20° C until measurements were taken. In addition, flies were used that came from a laboratory population that had been infested by mites, collecting and freezing all infected flies (n = 41) before the population was exterminated. Mites, which are easily visible on the ventral surface of the flies, were counted, and then the legs and/or wings of flies were removed and either mounted on slides or paper for later measurement. For laboratory flies wing length (WL) and fore-tibia length (FTL) were measured, and for the field collection WL and wing width (WW) were measured. In the field data set wing traits were used because the degree of wing asymmetry should strongly influence flight efficiency (in birds: Balmford et al. 1993), which in turn should be fitness related in free-living flies (Bennett and Hoffmann 1998). Additionally, wing FA and parasite associations have been reported in other Dipterans (Agnew and Koella 1997). Mite counts and FA measurements were performed by two different people.

Each trait was measured twice with an eyepiece graticule on a binocular microscope (×16). The asymmetry of each trait was the signed left-right difference. FA is the absolute value of this measure. A 2-way analysis of variance (ANOVA) performed on the repeated measures was used to assess whether asymmetry could be discerned from measurement error (as recommended by Palmer, 1994).

This is important because measurement error generates the same pattern of between sides error as FA. A significant side × individual association indicates FA was discernible from measurement error for all measures in both data sets (p < 0.008), and there was no directional asymmetry (the side effect was always NS; p > 0.14). One sample t-tests were additionally used to test for directional asymmetry and departures from normality (Palmer 1994). All measures were found to show FA (t-tests: all | t-values | < 1.63; p >0.11). Regression of absolute (unsigned) FA against mean trait size indicated FA was not associated with trait size, although it was close for WL in wild flies (WL, F_1,_74 = 3.33;*p*) = 0.072; All other F < 0.39; > 0.53). Therefore, a size corrected FA measure was not calculated, but wing length was included as a covariate in the absolute FA analyses. In addition, another measure of FA was calculated (FA5 Palmer, 1994: = Σ(R-L)^2^ /N), and a mean absolute FA was calculated for the traits under study as a composite measure of asymmetry (see Bennett and Hoffmann 1998; Martin and Hosken 2002). Analyses were carried out using all FA measures. All data were checked to fit the assumptions of parametric tests and transformed when necessary. However, it was not possible to completely normalise the distributions of absolute FA for WL or WW. In view of this, the residuals of the absolute FA MANOVA analysis were checked using Q-Q plots and Kolmogorov-Smirnov tests. There was some skew in both sets of residuals (*p* = 0.021 and 0.028 respectively). Nevertheless, since p-values were high, and analyses employing FA5 gave identical results, we are confident in the robustness of the results. Non-parametric tests were used whenever possible to avoid problems of non-normal data distribution, and these verify the general conclusions based on the multivariate parametric tests. Finally, due to missing data (i.e. for some flies wing width could not be accurately measured) sample sizes vary slightly.

## Results

### Longevity costs of mite infestation

**Figure 1.**
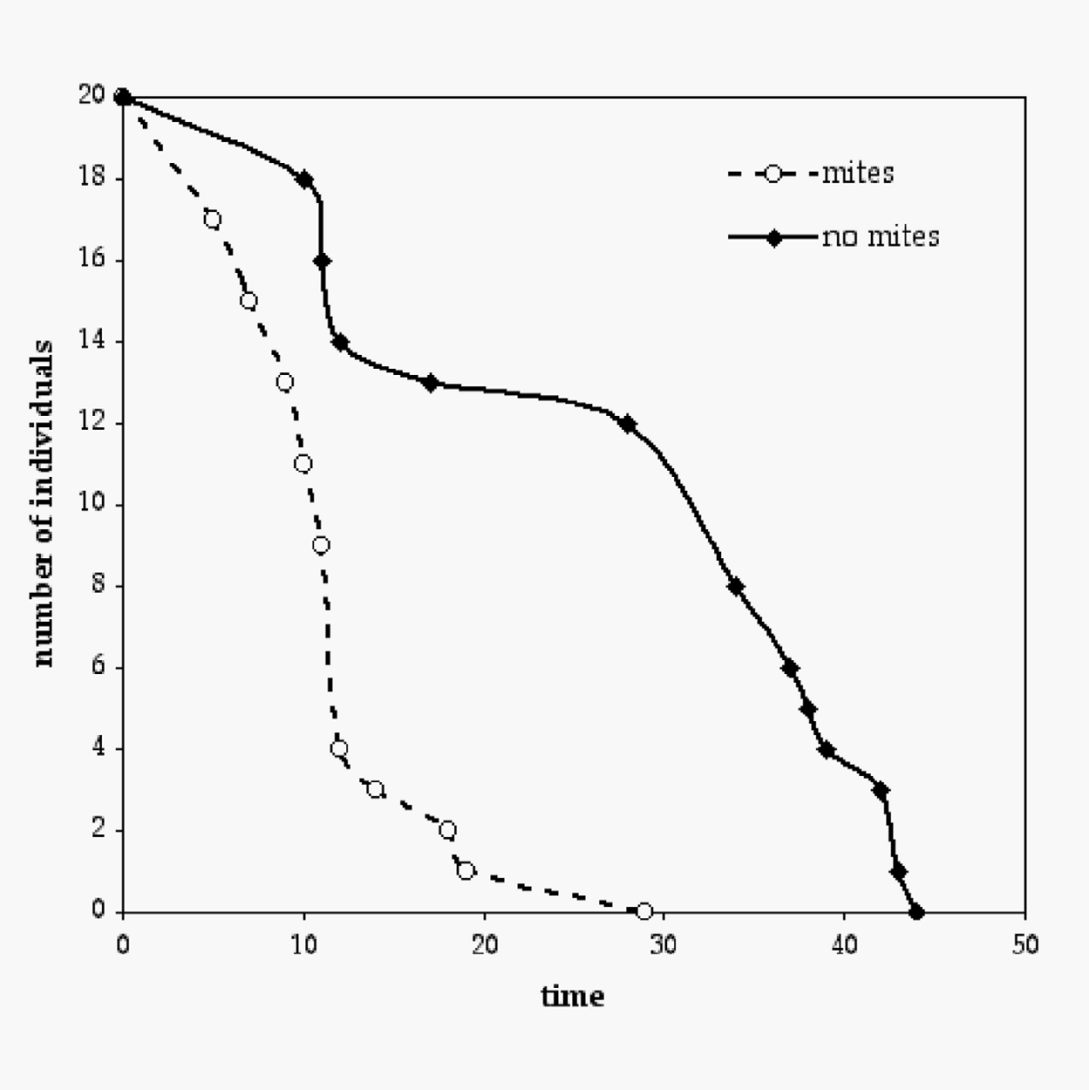
The cumulative survivorship curves for Sepsis *cynipsea* flies with or without *Pediculoides mesembnnae* mites. Initially there were 20 flies (10 females, 10 males) in each treatment, but mite infected flies died much faster. Time (the x-axis) is in days.

The impact of mites and mite number on longevity was analysed using Cox regression. Analysis with gender and presence of mites indicated that mite infestation significantly reduced longevity but there was no effect of gender (Cox regression: overall model c^2^ = 17.63, *df* = 2, *p* < 0.0001, *n* = 40; presence of mites: *p* < 0.0001, gender: *p* = 0.38, Figure 1). Inclusion of hind tibia length and number of mites generated similar results although neither covariate significantly affected survivorship (c = 17.92, *df* = 4, *p* = 0.0013, *n* = 40; presence of mites: *p*) = 0.0057, gender: *p* = 0.42, number of mites: *p* = 0.65, hind tibia length: *p*) = 0.75). These analyses indicate that mite infection significantly reduced male and female longevity and this effect was independent of the number of mites attached to an individual. It should also be noted that the number of mites used in this experiment (mean ± S.E. = 10 ± 2; range: min. 3, max. 27) was rather low and did not exceed numbers attached to field-caught flies. Therefore the negative impact on survivorship documented here was deemed to be a realistic and conservative (due to low mite numbers used) simulation of longevity effects of mite infestation in this species.

### Fluctuating asymmetry and mites

A multivariate general linear model of the larger field data set was performed with sex, body size and parasites as predictors and the two wing FA measures as the dependents. This indicated no associations between parasite number or body size and FA (F_2_,67 < 0.93; p > 0.4), but sex (male or female) had a significant multivariate effect (Wilk's Lambda F2,67 = 3.51; *p*) = 0.04). Univariate analysis revealed the sex effect was driven by WL FA (F_1_,72 = 7.22; *p*) = 0.009), with females being more asymmetrical than males, and there was no effect of sex on WW FA (F_1,_68 = 1.19; *p* = 0.28). Similarly, in analysis with mean wing FA as the dependent, only sex had a significant effect (sex, F_1_,68 = 6.33; *p*) = 0.01; WL F1,68 = 0.40; *p*) = 0.53; parasites, F1,68 = 0.44; *p*) = 0.51), with females again more asymmetrical in wing characters. Analyses with mite number as the dependent variable, likewise revealed no body size or asymmetry associations, and there was no sex difference in mite number (all p-values > 0.28). Essentially identical results were found using FA5 measures.

**Figure 2.**
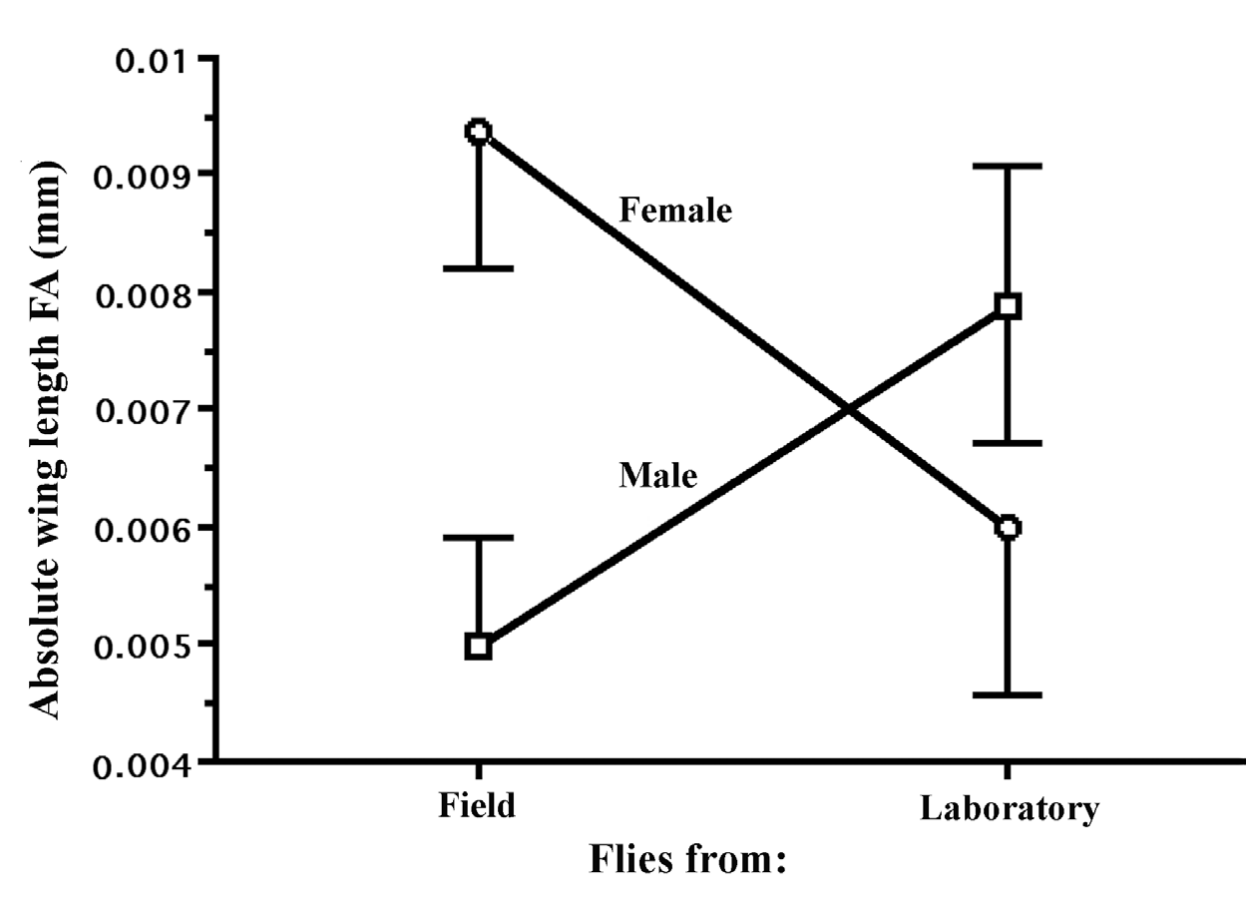
The interaction between sex and origin influencing Sepsis *cynipsea* wing length FA. Field females were more asymmetrical than field males and the reverse was true in laboratory-reared flies. Shown are means with standard errors.

To compare parasite numbers in laboratory and wild flies a general linear model was used with sex and fly origin (laboratory /field) as factors and wing length as a covariate. There were no significant effects or interactions (all F < 2.7; all p > 0.1; mean ±SE number of mites: laboratory = 9.7±8.9, wild = 13.1±14.3). Comparison of WL FA also revealed no parasite effect (F_1_,112 = 0.003; *p*) = 0.95) or size effect (F1,112 = 0.09; *p* = 0.75), but there was a significant sex by laboratory /wild interaction. Wild females tended to be more asymmetrical than wild males but the laboratory males were more asymmetrical (Figure 2; F1,112 = 5.83; *p* = 0.017). Non-parametric tests also indicated that FA and parasites did not vary between the laboratory and field (Kruskal-Wallis test: H < 3.45; > 0.17), and there was no association between WL FA and parasite number (Spearman Rank correlation: Z = 0.54; *p* = 0.59). When analyses were restricted to just the laboratory population, and included FTL FA, no significant associations were found between mite number and asymmetry (WL FA, F_1_,24 = 0.84; *p*) = 0.37; FTL FA, F1,24 = 1.67; *p*) = 0.21), and there was no effect of sex (F_1_,24 = 1.43; *p*) = 0.24). Identical results were obtained with FA5 and mean FA, and with the use of Spearman Rank Correlations. Finally, there was no association between absolute WL and WW FA in either parametric or non-parametric analyses (Spearman Rank correlation: Z = 0.43; *p* = 0.67), nor were absolute WL and FTL FA associated (Spearman Rank correlation: Z = 0.05; *p*) = 0.96). The repeatability of developmental stability for the laboratory flies (Whitlock 1998) was also calculated. Repeatability was moderate to low for all traits, especially wings (*R* FTL = 0.29; *R* hind tibia length = 0.37; *R* WL = 0.07).

One sample tests were used to compare the degree of FA in the wild-captured mite-infested flies with values previously published for mite-free, laboratory reared, flies (absolute FA as a % of trait size values were compared to avoid problems associated with measurer and measurement differences between studies). Regardless of whether parametric or non-parametric analyses were used, the only significant difference between the two studies was that mite infected males showed less WL FA (one-sample t-test; d.f. = 56; t = -3.79; *p* = 0.001: all other comparisons t <1.4; > 0.17) and this result remains significant after Bonferroni correction. Similarly with WL FA in laboratory flies (one sample sign test *p* = 0.049), but FTL FA did not differ between studies for either sex (all p > 0.08). Since FA did not scale with trait size, these analyses may be biased against the parasitized flies if they were larger (i.e. larger flies will have less FA as a % of trait size when FA scales with negative allometry relative to trait size or if it is size independent). However, comparison of hind tibia length, a measure of body size, between the studies indicated that the parasitized flies were smaller, and therefore should show larger % FA than the flies from Blanckenhorn et al.'s (1998) study, which they did not in any comparison.

## Discussion

Mites were costly to their hosts, causing a substantial decrease in fly longevity. This is consistent with many other host-parasite studies that document a range of costs to the host (Zuk 1987; Simmons 1990; Goater and Ward 1992; Goater et al. 1993; Yourth et al. 2002). For example, Polak (1996, 1997a) documents major fitness costs to a phoretic mite in another fly, *Drosophila nigrospiracula.* In spite of this however, no associations were found between parasites (presence or number) and FA in either laboratory or field flies. While the lack of statistical significance in the parasite-FA analyses could be due to Type II error, p-values are typically high, and sample size is large, exceeding that recommended by Palmer (1994). We are, therefore, reasonably confident in the robustness of the findings which suggest that there are no, or only very weak, associations between mites and FA. Because mites are known to attach to larvae (H. Ochs personal communication) and to attach to adult flies after eclosion, mite and host FA associations are expected. This is because if FA reflects general quality, and symmetrical hosts have superior immunity, symmetrical hosts will be less likely to come into contact with mites. Alternatively, mites might stress flies during development and directly cause increased FA. However, no support was found for any of these suppositions. This contrasts with findings in many other taxa. For example, FA and parasite load were positively related in male gobies (Sasal and Pampoulie 2000), mosquitoes harbouring microsporidian spores showed higher FA than controls (Agnew and Koella 1997), and Møller (1996b) reported that in most published studies FA was related to parasitism. Furthermore, it was suggested that FA might prove to be an indirect phenotypic marker of parasitism (Møller 1996b). Our results do not seem to support this idea. It may simply be that the most asymmetrical individuals harbouring most parasites suffer high mortality and are not sampled. However, in the more benign laboratory setting, no associations were seen either. Interestingly, the exceptions to the general pattern reported in Møller (1996b) were also insects, and for example, Ward et al. (1998) found no association between FA and parasitism in two species of cockroach infected with nematodes or in four grasshopper species infected with gregarine protozoans. Similarly, Polak (1993) found no association between FA and mite number in another fly (although nematode infection was associated with FA), and Yourth et al. (2002) find no associations between measures of host immunity (encapsulation response) and FA (but see, for example, Fair et al. 1999).

The FA comparison with previously published FA values from mite-free flies, revealed no significant differences in FA, except for WL FA, which was significantly lower in mite-infested males. This result remains significant with Bonferroni correction, and does not seem to be due to size correction effects since the flies from the previous study (Blanckenhorn et al. 1998) were larger and therefore, if anything, should have shown less FA as a proportion of trait size. This result appears to be counter to expectations and findings from many previous studies (reviewed in Møller, 1996b). For example, weta bush crickets parasitized by nematodes were more asymmetrical than those not parasitized, but as with our study, there was no association between FA and parasite size or number (Thomas et al. 1998). Again, one potential explanation that could reconcile this apparent discrepancy is that the most developmentally unstable male flies cannot cope with the additional stress of mites and die. However, the lack of concordance between FTL and wing FA comparisons is cause to question this conclusion. It may simply be that mites are not stressful and hence no relationship was seen. However, our longevity results refute this, and if nothing else, mites represent additional mass that has to be carried during flight. This is perhaps the reason for the wing/mite association. If so the lack of concordance between the various FA measures would explain why leg FA does not differ in flies with or without mites. However, even if this were so, why is the difference only manifest in males and not females, especially since females tend to be more asymmetrical than males in wing characters (this study and Blanckenhorn et al. 1998)? Additionally, mite number was not associated with any FA measure, so, on balance, the data suggest that the wing FA and mite association is not substantially important. Moreover, the general lack of FA difference between parasitized and non-parasitized flies appears to support the conclusion that FA and parasitic mites are not, or at best very weakly associated in these flies.

As previously reported (Allen and Simmons 1996), FA was not size dependent for any of the characters studied, even for FTL that is sexually selected. This is counter to suggestions that sexually selected traits should show a negative association between size and FA if they are to reliably signal an individual's quality. This lack of association is consistent with the parasite data however, which also indicates that FA is not a quality indicator. This is additionally in accordance with an increasing number of studies, which fail to find evidence for FA/good genes associations (Hunt and Simmons 1997; Blanckenhorn et al. 1998; Bjorksten et al. 2000; Blanckenhorn and Hosken 2003). Additionally, parasites have been reported to differentially increase levels of FA in sexually selected traits compared to general morphological characters (Møller, 1992b). However, we found no differences in FTL FA in mite-infected and non-infected flies, possibly because mites have attached to these flies after traits are fully developed. As stated above, and also reported previously (Blanckenhorn et al. 1998), female flies were more asymmetrical in WL than male flies in the field sample, but the interaction (Figure 1.) indicates this pattern is reversed in the laboratory. Why this occurs is unclear, but it may be related to higher predation on asymmetrical males in the field. Swaddle (1997) found that yellow dung flies, *Scathophaga stercoraria,* kill more asymmetrical *M. domestica* in a laboratory setting. *S. stercoraria* are, at times, found on the same dung pats as *S. cynipsea,* and certainly eat them in the laboratory (Mühlhäuser and Blanckenhorn 2002). Since male *S. cynipsea* spend more time on dung pats than females, a predation effect on FA is possible. Moreover, similar predation effects have been reported on free-living house flies (Møller 1996a). It is also possible that females with higher asymmetry are selected against in the laboratory. If females with higher FA were less able to escape male harassment in captivity because of reduced flight efficiency, then this would result in increased mortality for these females (Blanckenhorn et al. 2002; Mühlhäuser and Blanckenhorn 2002; Martin and Hosken 2003a,b). These explanations probably deserve further investigation. Nevertheless, this is one of a growing number of studies to find no concordance between FA of various traits within animals (Hunt and Simmons 1997; Hosken et al. 2000). This may simply be because measuring developmental stability with FA is imprecise (Whitlock 1996; Van Dongen 1998), but the lack of concordance potentially argues against FA as a general measure of genetic quality. Low FA repeatability was also evident, especially for wing length, which is consistent with the non-significant heritability of FA that is often reported (Blanckenhorn and Hosken 2003).

There were no differences in mite number between the sexes in spite of the sex differences in FA. Sex differences in immune function have been reported in insects (Kurtz et al. 2000), but there may be no differences in *S. cynipsea,* assuming mite infestation reflects immune system quality. No association was evident between mite number and body size. Studies on other insects have reported such an association presumably for purely mechanical reasons (Bonn et al. 1996).

In conclusion, we found no evidence for an association between mite number and FA in *S. cynipsea,* nor were mite-infected flies more asymmetrical than uninfected flies in spite of considerable longevity costs to infection. Additionally, there were no sex differences in mite infestation despite sex differences in FA. However, an interaction was found between fly origin and wing FA that may be indicative of differential costs to FA in the laboratory and field. These results indicate parasite/FA associations may not be as widespread as previously indicated.

## Abbreviations

**FA**: fluctuating asymmetry - small random deviations from perfect symmetry,
**FTL**: fore-tibia length,
**WL**: wing length,
**WW**: wing width
